# Sequential short-course radiation therapy and chemotherapy in the neoadjuvant treatment of rectal adenocarcinoma

**DOI:** 10.1186/s13014-019-1358-1

**Published:** 2019-08-19

**Authors:** Angela Y. Jia, Amol Narang, Bashar Safar, Atif Zaheer, Adrian Murphy, Nilofer S. Azad, Susan Gearhart, Sandy Fang, Jonathan Efron, Tam Warczynski, Amy Hacker-Prietz, Jeffrey Meyer

**Affiliations:** 1 Department of Radiation Oncology and Molecular Radiation Sciences, Johns Hopkins University School of Medicine, Baltimore, MD USA; 2 Department of Surgery, Johns Hopkins University School of Medicine, Baltimore, MD USA; 3 Department of Radiology and Radiological Science, Johns Hopkins University School of Medicine, Baltimore, MD USA; 4 Department of Oncology, Johns Hopkins University School of Medicine, Baltimore, MD USA

**Keywords:** Rectal cancer, Short-course chemoradiotherapy, Watch and wait

## Abstract

**Background:**

There is continued debate regarding the optimal combinations of radiation therapy and chemotherapy in the preoperative treatment of locally advanced rectal adenocarcinomas. We report our single-institution experience of feasibility and early oncologic outcomes of short-course preoperative radiation therapy (5 Gy X 5 fractions) followed by consolidation neoadjuvant chemotherapy.

**Methods:**

We reviewed the records of 26 patients with locally advanced rectal adenocarcinoma. All patients underwent short course radiotherapy (5 Gy X 5 fractions) followed by chemotherapy [either modified infusional and bolus 5-fluorouracail and oxalipatin (mFOLFOX6) or capecitabine and oxaliplatin] prior to consideration for surgery. A full course of chemotherapy was defined as at least 8 weeks of chemotherapy.

**Results:**

There were five clinical (c) T2, 16 cT3, and five cT4 rectal tumors, with 88% cN+. Twenty-five patients received a median of 4 cycles (range 3 to 8) of mFOLFOX6 (with one cycle defined as a two-week period); one patient received 3 cycles of capecitabine and oxaliplatin. All patients completed SCRT; 81% completed the full course of neoadjuvant chemotherapy with 19% requiring dose reductions in chemotherapy, most commonly due to neuropathy. Nineteen patients underwent post-treatment endoscopic evaluation, and nine patients were noted to achieve a complete clinical response (CCR). Six of the nine patients who achieved CCR opted for a non-operative approach of watch-and-wait. Twenty patients underwent surgical resection; pathologic complete response was observed in seven (35%) of these twenty. The main radiation-associated toxicity was proctitis with CTCAE Grade 2 proctitis observed in seven patients (27%). Post-operative Clavien-Dindo Grade 3 complications within 30 days of surgery were identified in six patients (30%), with no Grade 4 or 5 adverse events. Median length of hospital stay was 4.5 days (range 2–16 days); three patients were readmitted within a 30 day period.

**Conclusions:**

Short course preoperative radiotherapy followed by neoadjuvant chemotherapy was well-tolerated and achieved oncologic outcomes that compare favorably with short-course radiation therapy alone or long-course chemoradiotherapy. This regimen is associated with high rates of clinical and pathologic complete response.

## Background

Radiation therapy and chemotherapy are commonly used in the preoperative setting for selected patients with rectal adenocarcinoma. Radiation has been administered in short-course fashion, typically 25 Gy delivered in 5 fractions, or concurrently with chemotherapy in more protracted chemoradiotherapy regimens. The Dutch TME trial demonstrated that preoperative short-course radiation therapy (SCRT) reduced the risk of local-regional recurrence risk relative to surgery (total mesorectal excision) alone, and preoperative chemoradiotherapy courses are likewise associated with low rates of pelvic tumor recurrence following surgery [1, 2].

Despite reduction in local-regional recurrence risk with neoadjuvant treatments, distant disease recurrence remains a substantial risk for patients with locally advanced disease. In the German CAO/ARO/AIO-94 trial, patients treated with preoperative long-coursechemoradiotherapy, surgery, and adjuvant chemotherapy had a 7.1% incidence of local recurrence by 10 years, but a nearly 30% incidence of distant metastatic recurrence [2]. Emphasizing the paradigm of spatial cooperation, a recent trend in rectal cancer management has been to integrate systemic therapy into the neoadjuvant setting for patients with high-risk disease, ranging from a few cycles of chemotherapy to “total neoadjuvant” therapy [3, 4]. Various sequences and combinations of chemotherapy, radiation therapy, and chemoradiotherapy have been reported and are the subject of clinical trials. Of note, chemotherapy has demonstrated the ability to achieve profound local effects on gross primary rectal tumors, including induction of pathologic complete responses [5–8].

SCRT yields similar oncologic and toxicity outcomes relative to chemoradiotherapy for patients with rectal cancer, and SCRT with extended break prior to surgery is associated with reduced postoperative complications relative to SCRT with immediate surgery [9–11]. In a recent randomized trial evaluating patients with clinical stage T4 or fixed T3 rectal tumors, SCRT followed by consolidation chemotherapy prior to surgery yielded superior overall survival outcomes compared to chemoradiotherapy, albeit without significant differences in disease-free survival, nor local or distant disease control rates [12]. The phase III RAPIDO and STELLAR clinical trials are also evaluating SCRT and consolidation chemotherapy [13, 14]. Investigators from Washington University have also demonstrated the feasibility and potential oncologic benefits of SCRT and consolidation chemotherapy relative to chemoradiotherapy [15]. SCRT remains a relatively uncommon treatment regimen, relative to protracted chemoradiotherapy, in the United States [16].

In this report, we describe our institutional experience with preoperative SCRT and consolidation chemotherapy, with an emphasis on early oncologic outcomes (clinical and pathologic response rates), as well as treatment-associated toxicities, including details of post-operative morbidities. We also report early results of SCRT and consolidation chemotherapy leading to clinical complete response and subsequent non-operative/watch-and-wait management.

## Methods

### Patients

A retrospective review was conducted using data from Johns Hopkins Hospital between January 2017 to January 2019. Patients with clinically localized biopsy-proven rectal adenocarcinoma who were treated with SCRT and consolidation chemotherapy were included for analysis. Patients eligible for this treatment had clinical stage II or III cancer, with the distal edge of the tumor at 12 cm or less from the anal verge. All patients underwent MRI for local tumor staging, and all had CT imaging to evaluate for distant disease. Patients underwent radiation and surgery at our institution. This study was approved by our Institutional Review Board.

### Treatment

The SCRT was delivered prior to the chemotherapy course. For the radiation treatment planning, the gross tumor volume and regional lymphatic spaces, including the mesorectum and internal iliac regions, were identified as the clinical target volume with expansion to generate a planning target volume (PTV). Treatments were planned with either 3D conformal or volumetric modulated arc radiation therapy (VMAT). The prescription dose was 5 Gy X 5 fractions. There was no integrated boost or dose tiers. Cone beam computed tomography (CBCT) was used for image guidance. Treatments were delivered on consecutive weekdays, with plan to complete the radiation course within 5–7 days.

Consolidation chemotherapy began typically about 2 weeks following completion of the radiation course. Twenty-five patients received mFOLFOX6: infusional and bolus 5-fluorouracil (5-FU) and oxaliplatin at standard dosing of 5FU 400 mg/m2 bolus and 2400 mg/m2 over 46 h and oxaliplatin at 85 mg/m2 every 2 weeks, where one cycle was 2 weeks. One patient received capecitabine at 1500 mg twice daily on days 1–14, and oxaliplatin at 135 mg/m2 once every 3 weeks (one cycle was 21 days). Number of chemotherapy cycles and dose modifications were made at provider discretion.

Reevaluation for surgery followed the completion of chemotherapy, and typically entailed repeat endoscopy and imaging. Complete clinical response was defined as the absence of tumor on endoscopic evaluation and biopsy. The surgical resection followed the principles of total mesorectal excision. Postoperative chemotherapy was delivered at the discretion of the treating medical oncologist.

### Outcomes

Patient records were evaluated for treatment-associated toxicities, including surgical morbidities, as well as oncologic outcomes in the form of imaging treatment response and histopathologic outcomes from the surgeries. Post-treatment MRI tumor regression grade (TRG) and histopathology TRG were assigned [17, 18]. We calculated the neoadjuvant response (NAR) score as follows:

NAR = [5pN – 3(cT – pT) + 12]^2^/9.61, where pN = pathologic nodal stage, cT = clinical T stage, and pT = pathologic T stage [19].

Common terminology criteria for adverse events (CTCAE) v 4.03 was used to grade toxicities during the period from the beginning of radiation treatment to surgical reevaluation. Toxicities were reviewed retrospectively. Surgical adverse events, defined as occurring within 30 days in the post-operative setting, were graded according to the Clavien-Dindo classification, and were also retrospectively evaluated from patient records [20].

## Results

### Patient characteristics and neoadjuvant treatment

Twenty-six patients with rectal adenocarcinoma were reviewed in this study. Patient characteristics are show in Table 1. The majority of the patients (22) were treated with VMAT for the radiation course. Twenty-five of the 26 patients were treated with daily image guidance in the form of CBCT. All patients completed the SCRT course. Median time to completion of radiation therapy was 7 days (range: 5–11 days). Twenty-five of the patients received consolidation chemotherapy in the form of mFOLFOX6, with a median of 4 cycles (range: 3–8 cycles). One patient received 3 cycles of capecitabine and oxaliplatin. The median time from completion of SCRT to initiation of chemotherapy was 14.5 days (range: 7–44 days). The median time from completion of SCRT to either surgery or endoscopic evaluation (for the patients who were followed with watch-and-wait) was 14.5 weeks (range: 11.7–25.7 weeks).

**Table 1:** Patient Characteristics

Characteristics	*n* = 26
Gender	
Male	20 (77%)
Female	6 (23%)
Age (years)	
Median	52
Range	38–77
Clinical stage	
T2	5 (19%)
T3	16 (62%)
T4	5 (19%)
N1–2	23 (88%)
Distance (cm) from anal verge	
Median	7
Low (0–5)	10
Mid (> 5–10)	11
High (> 10–15)	5
Doses* of neoadjuvant chemotherapy	
Median	4
Range	3–8
Surgery	
Surgical resection	20 (77%)
Non-operative management/watch-and-wait	6 (23%)

*See text for definition of dose/cycle

### Treatment response

Post-treatment pelvis MRI was obtained in 23 of the 26 patients, at a median of 13.3 weeks (range: 9.6–18.9 weeks) from completion of SCRT. All patients who underwent post-treatment MRI were assigned a radiographic/MRI tumor regression grade. This result, the histopathologic outcomes of patients who went to surgery (including margin status), and the NAR scores for the individual patients are shown in Table 2.

**Table 2:** Treatment results

Patient	Clinical stage	Post-treatment MRI TRG	Post-treatment endoscopy response	Time to surgery (weeks)	Pathologic stage	Radial Margin	Pathologic TRG	NAR
1	T2 N1	3	No post-treatment endoscopy	18.1	T3 N1	Negative	Not assessed	41.6
2	T2 N1	–	Complete	–	–	–	–	N/A
3	T2 N2	3	No post-treatment endoscopy	12.9	T2 N0	Negative	1	15
4	T2 N2	1	Complete	–	–	–	–	N/A
5	T2 N2	3	Not complete	25.7	TisN0	Negative	0*	0
6	T3 N0	2	No post-treatment endoscopy	17.3	T0 N0	Negative	0	0.9
7	T3 N0	2	Complete	–	–	–	–	N/A
8	T3 N1	–	No post-treatment endoscopy	12.3	T0 N0	Negative	0	0.9
9	T3 N1	2	Not complete	13.0	T3 N0	Negative	1	15
10	T3 N1	2	Complete	16.9	T3 N1	Negative	1	30.1
11	T3 N1	2	Not complete	13.6	T0 N0	Negative	0	0.9
12	T3 N2	2	Not complete	23.4	T3 N2	Positive	3	50.4
13	T3 N2	3	Not complete	17.9	T1 N0	Negative	2	3.7
14	T3 N2	3	Not complete	17.3	T1 N0	Negative	3	3.7
15	T3 N2	1	Not complete	13.6	T0 N0	Negative	0	0.9
16	T3 N2	3	No post-treatment endoscopy	14.1	T2 N1	Negative	2	20.4
17	T3 N2	1	Complete	–	–	–	–	N/A
18	T3 N2	2	No post-treatment endoscopy	13.9	T0 N0	Negative	0	0.9
19	T3 N2	2	Complete	24.3	T0 N0	Negative	0	0.9
20	T3 N2	3	Complete	18	T3 N1	Negative	2	30.1
21	T3 N2	3	Not complete	13.6	T3 N1	Negative	2	20.4
22	T4bN0	1	Complete	–	–	–	–	N/A
23	T4aN1	–	No post-treatment endoscopy	13	T0 N0	Negative	0	0
24	T4aN2	3	Not complete	13.9	T3 N1	Positive	2	20.4
25	T4aN2	3	Not complete	13.1	T3 N1	Negative	2	20.4
26	T4bN2	3	Complete	–	–	–	–	N/A

*Abbreviations: TRG* treatment response grade, *CCR* clinical complete response, *NAR* neoadjuvant response score

Note: Patients without pathology assessment were those followed with watch-and-wait approach. Pathologic tumor regression of 0 represents pathologic complete response. *The patient with pTis disease is assigned a score of 0

Nineteen of the 26 patients were re-evaluated with endoscopy prior to surgery. A total of 9 patients achieved complete clinical response (CCR) on endoscopy. Six of these patients elected for watch-and-wait/non-operative management after consultation with the colorectal surgeon. Patients on watch-and-wait have been followed for a median of 18 weeks (range: 2.7–42 weeks). One patient followed on watch-and-wait developed local recurrence of disease at 20 weeks from CCR on endoscopy.

Of the 20 patients who proceeded to surgery, pathologic complete response (pCR) was achieved in 7 (35%). One patient had pTis disease. For the 20 patients, the NAR score was low in 10 (50%), intermediate in 2 (10%), and high in 8 (40%). One patient developed local recurrence of cancer, at 40 weeks following initial surgery.

Additional chemotherapy following CCR (non-operative management) or surgery was administered at the discretion of the treating medical oncologist. Three of the six watch-and-wait patients continued to receive chemotherapy after achieving CCR. Fourteen of the 20 patients who underwent surgery received post-operative chemotherapy.

### Toxicities

The main radiation-associated toxicity was proctitis, which reached CTCAE grade 2 level in 7 (27%) of the 26 patients and typically developed shortly after completion of the SCRT course. Eighty-one percent of the patients were able to complete the full intended course of consolidation chemotherapy, with 19% requiring dose reductions, most commonly relating to neuropathy (60%). Post-operativeClavien-Dindo Grade 3 complications within 30 days of surgery were identified in six patients (30%), with no Grade 4 or 5 adverse events. These events included bleeding episodes requiring re-operation, ostomy revision, and abscess formation requiring intervention. There were no grade 4 or 5 events. Median hospital stay following surgery was 4.5 days (range: 2–16 days). Three of the patients undergoing surgery were readmitted to the hospital within 30 days of discharge.

## Discussion

In addition to treating micrometastatic systemic disease, chemotherapy is capable of inducing substantial downstaging effects on primary rectal tumors, including disease eradication to pCR status [5–8]. The TIMING trial showed a relationship between increasing the number of consolidation neoadjvuant chemotherapy cycles with higher pCR rates in patients with rectal cancer in a cohort of patients treated with neoadjuvant chemoradiotherapy [21]. Local treatment response as measured by downstaging and pCR rates may be maximized with a combination of radiation therapy- SCRT or long-course chemoradiation- and systemic therapy, to accompany the potential systemic control benefits of chemotherapy. Details of the optimal combinations remain to be determined.

The neoadjuvant rectal (NAR) score is a function of the post-neoadjuvant treatment pathologic nodal stage as well as the T downstaging of the primary tumor (cT – pT). It was validated as a surrogate for overall survival using data from the NSABP R-04 study, where a low score was associated with improved overall survival. The NAR has not been validated in patients receiving SCRT and consolidation chemotherapy, but we have reported it as another potentially important metric for comparison with long-course chemoradiotherapy outcomes. In our study, 60% of the patients had low or intermediate NAR scores, although this percentage is likely an underestimate of the true effect of this treatment approach, as the six patients who reached CCR and were followed were not included in the calculation.

Treatments that induce high CCR/pCR rates will also be necessary for more patients to be able to be managed with non-operative/watch-and-wait approaches, allowing for possible organ preservation, another emerging trend in rectal cancer management. Recent studies have demonstrated that many selected patients with CCR following neoadjuvant therapy have durable local control (without surgery) to the intermediate term [22, 23]. Of note, almost all watch-and-wait series to date have reported on patients treated with chemoradiotherapy, rather than SCRT. Bujko et al. reported on 30 patients treated with SCRT (without consolidation chemotherapy) [24]. Six of the patients achieved CCR after a median initial time to evaluation of 10.3 weeks. One of the 6 patient had local relapse of disease. Our report adds data to this early experience. Further follow-up will be necessary to ensure the durability of disease control in patients who reach CCR and are followed without immediate surgery.

SCRT is well established as a neoadjuvant treatment for rectal cancer. SCRT was the backbone for the Swedish and Dutch trials that demonstrated radiation to be an important neoadjuvant treatment modality by way of reduction of local-regional recurrence risk relative to surgery alone [2, 25]. Two randomized trials showed neoadjuvant SCRT and chemoradiotherapy to have similar oncologic outcomes, including long-term pelvic control, as well as similar late toxicity profiles, in patients with operable rectal cancer, further validating SCRT as an appropriate radiation approach [9, 10]. Other studies have helped establish SCRT and consolidation chemotherapy as a seamless sequential treatment for patients with rectal cancer. SCRT followed by chemotherapy is the experimental arm in the RAPIDO and STELLAR randomized clinical trials [13, 14].

SCRT has not been widely adopted in US centers, where long-course chemoradiotherapy is the most common neoadjuvant treatment. Investigators from Washington University have reported on SCRT and SCRT followed by consolidation chemotherapy, with promising results [15, 26, 27]. A study by Markovina et al. compared SCRT followed by 4 cycles of FOLFOX with matched patients receiving chemoradiotherapy without consolidation chemotherapy but treated with postoperative chemotherapy [15]. Overall survival and local control were similar between the two groups, but distant metastasis free-survival and disease-free survival favored the SCRT/chemotherapy group (3-year disease-free survival 85% versus 68%, *p* < 0.05).

In our institutional experience, a regimen of SCRT and approximately 2 months of consolidation chemotherapy was well tolerated and led to high rates of tumor downstaging, CCR and pCR rates, and reasonable post-surgical morbidity outcomes. The high pCR rate possibly reflects the contribution of the chemotherapy, for aforementioned reasons, as well as the effect of an extended time period from completion of radiation to surgery. Notably, this extended period did not significantly adversely impact patient tolerance to surgery in our series.

There a number of limitations to our report. There was some degree of heterogeneity in the administered consolidation chemotherapy courses, and post treatment evaluations were not standardized. Toxicity outcomes were retrospectively reviewed. In addition, there was not sufficient follow-up to evaluate long-term toxicities and oncologic outcomes, including disease-free and overall survival. Our focus was on short-term oncologic and toxicity outcomes.

## Conclusion

We report a single-institution experience of sequential SCRT and consolidation chemotherapy in the preoperative treatment of rectal cancer. This treatment course was well-tolerated and achieved early oncologic outcomes (downstaging and pCR rates) that compare favorably with results from neoadjuvant chemoradiotherapy series. A number of clinically relevant questions remain to be addressed, including the timing of the neoadjuvant chemotherapy (prior to or after the radiation therapy), the issue of chemoradiotherapy versus SCRT, the amount of chemotherapy to be delivered prior to surgery, and whether or not there is a role for post-operative chemotherapy in patients treated with this general approach. Ongoing studies will further define the best means of integrating radiation therapy and chemotherapy in the management of rectal cancer, with the ultimate goals of optimizing both local-regional and distant disease control.

## Data Availability

All data generated or analysed during this study are included in this published article.
